# Transcriptome from Paired Samples Improves the Power of Comprehensive COVID-19 Host-Viral Characterization

**DOI:** 10.3390/ijms241713125

**Published:** 2023-08-23

**Authors:** Ognjen Milicevic, Ana Loncar, Dzihan Abazovic, Marija Vukcevic, Dragana Despot, Tatjana Djukic, Vladimir Djukic, Andjela Milovanovic, Nikola Panic, Nemanja Plecic, Ana Banko

**Affiliations:** 1 Institute for Medical Statistics and Informatics, Faculty of Medicine, University of Belgrade, 11000 Belgrade, Serbia; ognjen011@gmail.com; 2 Institute for Biocides and Medical Ecology, 11000 Belgrade, Serbia; dranaloncar@gmail.com (A.L.); marija.vukcevic@biocidi.org.rs (M.V.); drdespot@hotmail.com (D.D.); 3 Biocell Hospital, 11000 Belgrade, Serbia; adzihan@gmail.com; 4 Institute of Medical and Clinical Biochemistry, Faculty of Medicine, University of Belgrade, 11000 Belgrade, Serbia; tatjana.djukic@med.bg.ac.rs; 5 Faculty of Medicine, University of Belgrade, 11000 Belgrade, Serbia; office@dragisamisovic.bg.ac.rs (V.D.); andjela.milovanovic@ymail.com (A.M.); nikola.panicmail@gmail.com (N.P.); 6 University Clinic “Dr Dragisa Misovic”, 11000 Belgrade, Serbia; plecicnemanja94@gmail.com; 7 Clinic for Medical Rehabilitation, Clinical Center of Serbia, 11000 Belgrade, Serbia; 8 Institute of Microbiology and Immunology, Faculty of Medicine, University of Belgrade, 11000 Belgrade, Serbia

**Keywords:** transcriptome, metagenomics, COVID-19, therapeutics, pairing, differential expression (DEG), SARS-CoV-2, RNA

## Abstract

Previous transcriptome profiling studies showed significantly upregulated genes and altered biological pathways in acute COVID-19. However, changes in the transcriptional signatures during a defined time frame are not yet examined and described. The aims of this study included viral metagenomics and evaluation of the total expression in time-matched and tissue-matched paired COVID-19 samples with the analysis of the host splicing profile to reveal potential therapeutic targets. Prospective analysis of paired nasopharyngeal swabs (NPS) and blood (BL) samples from 18 COVID-19 patients with acute and resolved infection performed using Kallisto, Suppa2, Centrifuge, EdgeR, PantherDB, and *L1000CDS2* tools. In NPS, we discovered 6 genes with changed splicing and 40 differentially expressed genes (DEG) that yielded 88 altered pathways. Blood samples yielded 15 alternatively spliced genes. Although the unpaired DEG analysis failed, pairing identified 78 genes and 242 altered pathways with meaningful clinical interpretation and new candidate drug combinations with up to 65% overlap. Metagenomics analyses showed SARS-CoV-2 dominance during and after the acute infection, with a significant reduction in NPS (0.008 vs. 0.002, *p* = 0.019). Even though both NPS and BL give meaningful insights into expression changes, this is the first demonstration of how the power of blood analysis is vastly maximized by pairing. The obtained results essentially showed that pairing is a determinant between a failed and a comprehensive study. Finally, the bioinformatics results prove to be a comprehensive tool for full-action insights, drug development, and infectious disease research when designed properly.

## 1. Introduction

During the current coronavirus disease-19 (COVID-19) pandemic, over 768 million infected people were registered, of which even 6.9 million ended fatally, https://www.who.int/ (accessed 18 June 2023). Accomplishing scientific achievements, such as pathogen identification and vaccine synthesis, were priority goals due to the heavy burden on health systems and the economy. Still, further efforts are necessary to understand the complex interactions between the host and severe acute respiratory coronavirus 2 (SARS-CoV-2). Although COVID-19 apparently shares numerous clinical manifestations with other common respiratory viral infections, a significant peculiarity of SARS-CoV-2 infection has been observed over time [1]. Firstly, many studies have proven that SARS-CoV-2 has the capacity to infect different human cell types managing to successfully evade the host immune response [2]. Key features of the innate immune functions disturbance include unrecognizable viral replication, dysregulation of interferon response and/or signaling pathways, exhaustion and reduction of lymphocytes, particularly CD4+ T, CD8+ T, and natural killer (NK) cells, and finally, development of hyperinflammation and even cytokine storm [3,4,5,6]. All listed and described features could lead to multiple-organ dysfunction syndrome (MODS) and acute respiratory distress syndrome (ARDS), a major cause of death in COVID-19 [7]. However, while the evolution of SARS-CoV-2 continuously leads to the generation of new variants and subvariants, and the rate of infections is simultaneously increasing, the spectrum of clinical manifestations and risk factors for adverse events are also changing [8].

The integrity of the nasal microbiome and virome may also be disturbed by an exogenous infective stressor such as SARS-CoV-2, leading to upregulation of the host innate immune system, activation of dormant pathogens, and establishment of microbial nasal mucosal host gene patterns [3,9]. To date, knowledge about these interactions is lacking not only in the field of COVID-19 but also in other infectious diseases. Due to limited methodological possibilities of targeted sequencing on cultures, next-generation sequencing (NGS)-based methods have opened up a wide range of possibilities for understanding metatranscriptomics. However, NGS still could not be widely used because of the complex and time-consuming execution, cost prohibition, demanding quality and sample volume, and difficulties in implementation to accurately and comprehensively describe the entire virome and microbiome together with the host response [10]. Therefore, the optimization of new methods and computational workflow to simultaneously characterize virome, microbiome, and host genome directly from a variety of clinical samples is expanding [10,11,12].

As a primary site of infection pathogenesis, local nasal expression is more COVID-19-dependent and specific. So far, transcriptome profiling studies have shown significantly upregulated genes and biological pathways altered during acute infection, such as proinflammatory cytokines, chemokines, enzymes in neutrophil-mediated immunity, and several IFN-stimulated genes [13,14,15,16]. However, data showing experimental validation, potential diagnostic use, and detailed characterization of variations with age, sex, disease period, and/or severity are generally scarce [17]. On the other hand, a systemic expression derived from whole blood could be more relevant to reflect the virus-induced host immune system imbalance. Thus, previous research conducted on blood samples from severe COVID-19 patients showed altered gene expression associated with inflammatory and hypercoagulability pathways, as well as elevated neutrophil activity and expression of coagulation and platelet function genes [18,19].

It could be observed that previous research has not yet examined and described changes in the transcriptional signatures during a defined time frame. This, in particular, implies considering paired samples related to acute and resolved infection because otherwise, the interpretation of human expression may vary [20]. Moving toward a more comprehensive analysis of the host-viral relationship, it is of vital interest that the aims of this study include evaluating the expression in paired COVID-19 samples across different relevant tissues. The benefit of pairing the samples and eliminating inter-individual variation is quantified whenever possible. Moreover, this study analyzes the host splicing profile showing potential therapeutic targets.

## 2. Results

### 2.1. SARS-CoV-2 Dominates the Metagenomics Findings in Both Case and Control Cohorts

Metagenomic alignment of 36 nasopharyngeal swab samples yielded a total of 3772 species, out of which 2699 had unique mappings. After the analysis of unique reads, only 3 species that belonged to different assemblies of SARS-CoV-2 and SARS-CoV were statistically significantly different between cases and controls (Ensembl IDs 9000092, 9000103, 694009). Considering all reads (unique and multi-mapped), no individual species showed statistically different abundances. Still, the cases’ mean log-transformed value was bigger (0.008 vs. 0.002, *p* = 0.019), indicating a significant reduction in the viral RNA abundance at the control stage. Summation was attempted for all the phages and multiple bacterial taxa, but no significant differences were achieved.

The same analysis was performed on 12 pairs of blood samples yielding a total of 1794 species, out of which 1162 had unique mappings. There were no statistically significant differences in individual or summed categories for all or unique reads.

### 2.2. DEG Analysis in Blood Rescued by Pairing

DEG analysis for NPS and BL samples was repeated with both paired and independent designs, yielding a different result. The independent design of blood samples gave only two genes that were statistically significant, so this analysis was deemed unsuccessful and was not further processed. Conversely, paired design for BL samples yielded 78 significant DEGs with only 3 (3.8%) downregulated (Table 1). NPS samples yielded a comparable number of significant genes regardless of pairing—53 genes in an unpaired (Supplementary Table S1) mode and 40 genes in paired mode (Table 2), with an overlap of 23 genes. Of those 23 upregulated genes, the majority were interferon-regulated genes. Among prominent ones are CXCL10, a marker of acute viral infection, ISG15, both an extracellular cytokine and an intracellular protein modifier, IFI6 that may be involved in the regulation of cell apoptosis and IFI27, a proven predictor for COVID-19 outcomes. The upregulation was found in major histocompatibility complex genes, HLA-DR and HLA-A. Downregulation was noticed in only 2 genes in the unpaired design. The asymmetry in the regulation direction can be explained by the significant immune activation across the transcriptome resulting mostly in upregulation.

**Figure 1:**
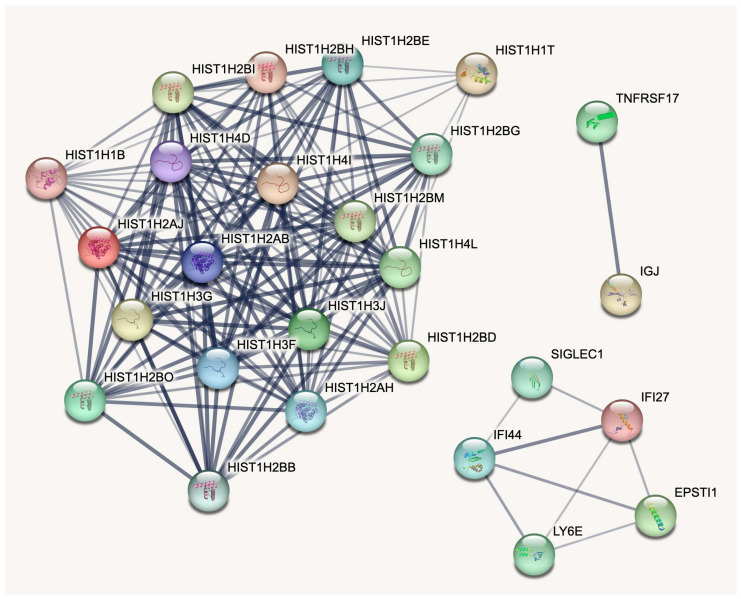
String database protein–protein interactions. The nodes represent genes, while the line width represents the strength of interaction. There are three clusters of genes without interconnections—nineteen histone genes, an immunity-related cluster with five genes, and the smallest cluster with two interacting genes.

Based on protein–protein interactions, there are three clusters of genes without interconnections (Figure 1). The largest cluster consists of 19 histone genes with abundant mutual connections between the members. The immunity-related cluster has 5 genes—IFI27, IFI44, LY6E, EPSTI1, and SIGLEC1. The smallest cluster has only two interacting genes—IGJ and TNFRSF17.

### 2.3. Pathway Analysis Confirms Known Disease Aspects and Reveals New Patterns

For the three successful design-tissue combinations (blood paired, nasopharyngeal paired, nasopharyngeal independent), we obtained significant results of pathway analysis for three ontologies—cellular components, biological processes, and molecular functions. The potentially asymmetric upregulation at the individual gene level is validated at this step. Molecular function pathways for BL samples (Table 3) show clinically expected downregulation in olfactory receptor activity, odorant binding, oxygen and heme binding, etc. The expected upregulation is mostly found in immune pathways (immunoglobulin receptor binding and antigen binding) and RNA metabolism (RNA binding and structural constituent of ribosomes), reflecting the infection mechanism of an RNA virus. Analysis of NPS with the same ontology gives reduced olfactory and G-protein coupled receptor activity, with the immune component shown as increased cytokine receptor binding (paired design) and antigen binding (unpaired design). The other tissue-ontology combinations show analogous upregulation involving multiple cell structures (Supplementary Tables S2–S9).

### 2.4. Non-Histone Gene Signatures Are Successfully Negated by L1000 Perturbagens

**Figure 2:**
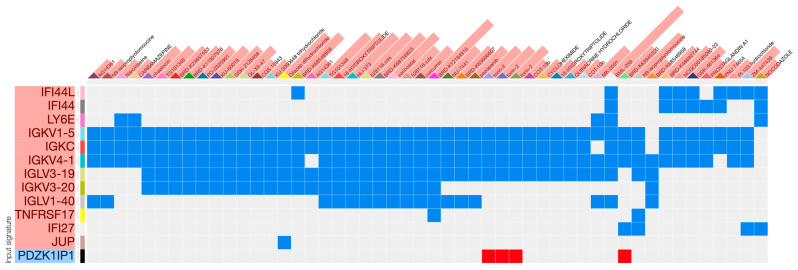
Hierarchically clustered heatmap of overlaps for individual perturbagens. Most substances match the genes from the immunoglobulin superfamily. Red genes are upregulated, and they are paired with blue perturbagen signatures of opposite sign. Opposite logic is valid for the downregulated PDZK1IP1 gene.

Comparison of the gene expression patterns with known perturbagens in L1000 gave the top-ranking substance BML-259 a score of 0.24. After analysis of the individual overlaps of substances, almost none of them included genes from the histone family. Because our programmatic annotation failed to recognize 14 of 20 histone genes (HIST*) in our list, we suspected a versioning annotation problem and repeated the analysis without the histone genes. The top-ranking substance remained the same, but the score increased to 0.47 (Table 4), and the best combination of BRD-K12184916 and Nocodazole had a score of 0.65 (Table 5). Nocodazole ranked 34th on the single substance list but appeared in 8 out of 12 top-ranking pairs and included the gene IFI27 with the lowest *p*-value. Clustered visualization shows that most single substances primarily affect the genes from the immunoglobulin superfamily (IG*) (Figure 2).

### 2.5. Alternative Splicing Changed at Both Event and Isoform Level

AS analysis events and isoforms in the independent design for two types of tissues are shown in Table 6. NPS samples show three genes with differentially spliced transcripts and three genes with differentially spliced events, with DCUN1D3 appearing in both groups. BL samples show thirteen genes with differentially spliced transcripts and three genes with differentially spliced events without mutual overlap. There were no overlapping genes between NPS and BL samples.

## 3. Discussion

To date, published studies have focused only on local nasal or systemic gene expression in acute COVID-19. It was unknown if there were any changes when the acute illness resolved. Taking this hypothesis into account, we examined gene expression profiles in paired designed samples of total RNA in acute and resolved SARS-CoV-2 infection to assess complete characterization and time-related differences of host-viral relationship in COVID-19 patients. The pathways that were mostly enriched were largely ones that control infection and inflammation, smell function, as well as a major portion of the receptor signaling and oxygen binding pathways. Besides identified biomarkers, this study revealed new drug co-targeting pathways.

Considering that the blood transcriptome reflects signals from different tissues and host biochemical processes, dispersion in RNA quantification could weak and mask many of those tissue-specific signals [21]. Moreover, individual variability influences the capacity to identify the processes primarily located in the blood, such as immune response pathways [17]. Thus, a larger sample size and additional standardizations in biological sampling are required. In our study, the blood samples would yield no usable results after removing the failed samples. On the other hand, nasopharyngeal swabs seem relatively robust towards the individual variability—the smaller set of genes is expressed in differentiated tissues such as nasal mucosa, thus increasing the number of transcripts per gene and the power of the study.

Using sample pairing design in this study increased the number of significant genes from 2 to 78, enabling a meaningful pathways analysis and functional/clinical validation of the project. Although this design represents a simple method for increasing power when interpersonal variability is large, genomic studies rarely take advantage of it when it comes to transcriptome analysis. Single-cell sequencing could be done at multiple time points to characterize cells in both time and space, but we strive to measure the improvement when applied in bulk sequencing for the characterization of systemic effects [22]. The pairing of samples does require repeated visits, patient tracking, and follow-up. Additionally, it is easier to find healthy controls for infectious diseases characterized by a complete resolution, while chronic infections with pathogen persistence or disease progression do not allow sampling due to the lack of a clear healthy control phase [23]. Regardless of the impossibility of obtaining RNA biomarkers of a specific disease, it is still possible to sample the RNA at two distinct time points and evaluate biomarkers of disease progression instead of the disease itself. Therefore, we emphasize that the gain in statistical power could be utilized in multiple fields and clinical problems.

The enrichment analysis has shown down-regulation of olfactory receptor, G protein-coupled receptor activity, and up-regulation of cytokine receptor binding in the nasal epithelium of paired samples, of which the first two were also down-regulated in paired blood samples. Olfactory dysfunction is a common symptom experienced by almost 53% of COVID-19 patients and affects approximately 7% of the COVID-19 convalescents [24,25]. There are several proposed mechanisms for SARS-CoV-2-related hyposmia/anosmia, one of them being T-cell–mediated inflammation persistent in the olfactory epithelium and the associated decline in the number of olfactory sensory neurons (OSNs) [26,27,28]. The exact mechanism of OSN decline is still not clear. The cilia of the OSNs have G-protein-coupled olfactory receptors involved in the transduction of extracellular signals through second messenger cascades controlled by heterotrimeric guanine nucleotide-binding proteins [29]. It is tempting to speculate that found down-regulation of olfactory receptor and G protein-coupled receptor activity may initiate the olfactory dysfunction in COVID-19 patients. Further, persistent viral infection could also drive ongoing damage to OSNs [30]. Indeed, the evidence for active SARS-CoV-2 infection was found in all of our nasopharyngeal swabs, although the cases’ mean log-transformed value was bigger (0.008 vs. 0.002, *p* = 0.019), indicating a significant reduction in the viral RNA abundance at the control stage.

Transcriptionally, our samples are characterized by the enrichment of pathways involved in cytokine receptor binding in nasal epithelium. After the appearance of SARS-CoV-2, cytokine storm was indicated as a main pathogenetic factor in COVID-19 [31]. A large number of studies have shown that COVID-19 patients have raised levels of several inflammatory cytokines, including IL-1β, IL-2, IL-6, IL-10, IFN-γ, TNF-α, IFN-γ-inducible protein 10 (IP-10), granulocyte macrophage-colony stimulating factor (GM-CSF), and monocyte chemoattractant protein-1 (MCP-1), that could be identified as the indicators of disease progression [32,33,34].

IFI27, interferon alpha inducible protein 27, transcription has recently been a proven predictor for COVID-19 outcomes [35]. A higher level of IFI27 with a lower level of DCUN1D3 is said to increase the risk for COVID-19 [36]. Earlier, it was also known as a biomarker for discrimination between influenza and bacteria in patients with suspected respiratory infection [37]. In our study, regardless of the type of sample, this was the strongest upregulated gene in acute COVID-19. These data, together with previously published studies, suggest that prognostic biomarkers targeting the family of IFI27 genes could potentially replace conventional diagnostic tools [35]. It could be of particular importance in future virus pandemics because IFI27 expression appears to be specific to viral illness [38]. Moreover, as the kinetics of IFI27 expression are poorly understood, the unresolved dilemma reported by other researchers was whether serial measurement of IFI27 expression would be more informative than a single time point measurement [35]. In order to resolve those dilemmas, genomic patterns from double-paired samples were, for the first time, comprehensively analyzed in our work. In this way, our results confirmed the hypotheses of previous authors.

Viruses manipulate cell cycle progression to generate resources and conditions favorable for viral production. Still, the effect of SARS-CoV-2 on cell cycle progression remains largely unknown [39]. Therefore, the identification of novel drug targets is an essential puzzle of comprehensive research. In the present study, we aimed to identify gene expression patterns with both single and co-targeted highly potent therapeutics. The L1000 perturbagens analysis showed BML-259, a potent cyclin-dependent kinase 5 (CDK5) inhibitor, as the top-ranking drug for acute COVID-19. As CDKs are key regulators of cell cycle progression, they represent promising therapeutic goals for cancer and neurodegenerative diseases. CDK has also been the target of various viral infections such as HIV, Herpes simplex virus, Zika, and Hepatitis B viruses, where its expression is altered in the affected cell [40]. Interestingly, when it comes to potential COVID-19 treatment, most literature data have been focused on CDK2, but not CDK5, identified by our study [40,41].

The greatest significance of our survey is that it reveals new co-targeting pathways of high potential for the success of synergistic drug action of nocodazole and BRD-K12184916. Even 65% of genes of acute COVID-19 patients are included in described co-targeted pathways: microtubule cytoskeleton organization and condition of hypoxia. These gene co-targets could serve as a promising step toward the identification of additional drug options, especially because some of them are already in use in other indications. The cytoskeleton, in particular, microtubules, have an essential role in cell mitosis, organization of cytoplasm, and controlling of cell movement, cell signaling, and trafficking of organelles. Their disruption leads to cell cycle arrest and loss of cellular architecture [42]. Therefore, various microtubule inhibitors, especially those that lead to cell arrest and apoptosis, such as nocodazole, have been used in the treatment of malignancies to synchronize cell proliferation. Moreover, there are indications that this mechanism could also be used for the purpose of antiviral action in human infections caused by West Nile virus, Cytomegalovirus, and even SARS-CoV-2 [43,44,45]. Inhibitors of the PI3K/AKT/mTOR signaling pathway, such as dactolisib, have potential antineoplastic activity targeting tumor cell apoptosis and growth inhibition in PI3K/mTOR-overexpressing tumor cells. In addition, those inhibitors have been shown as novel candidates to treat pathologic hypoxia that occurs in most human solid tumors [46]. Finally, even without these relatively new anti-hypoxia theories, those inhibitors have also been investigated in COVID-19. As they could upregulate IFN-induced antiviral responses, research was focused on reducing the COVID-19 severity and use in COVID-19 post-exposure prophylaxis in elderly patients [47,48].

The main limitation of the study was the sample size and the lack of clinical metadata that would enable further clinical correlations and stratification with transcriptomic biomarkers. The sample size was sufficient for the paired analysis performed without additional covariates but would be insufficient for investigating a subsample defined with the additional clinical data. As a consequence, even with strong statistical signals arising from the transcription of individual genes such as IFI27, we were unable to extrapolate their prognostic value to a broader clinical context. On the other hand, the study managed to unify the molecular mechanisms regardless of the differences in the clinical presentation of the disease, which could be considered a result that overcomes the described limitations.

Metagenomics analyses from the total RNA are susceptible to bias from existing fragments and show positive results even when conventional testing methods give a negative signal. However, the coexistence of other RNA viruses was not shown during the active disease or in the convalescence period.

Designing genomic research on a relatively novel subject harbors difficulty in formally estimating the required sample size and the proper design since maximizing the study power increases the probability of its success. Nasopharyngeal swabs are viable targets for both host and viral expression studies. Although they do not require sample pairing, those samples give limited insight into the systemic events. On the other hand, the analysis of blood samples benefits immensely when a sample pairing strategy is applied. In particular, the unpaired expression analysis performed in our research essentially failed to pinpoint significant changes, but the pairing successfully elucidated different pathways as well as the potential therapy targets with high concordance to clinical insights. Thus, the obtained results essentially showed that pairing is a determinant between a failed and a comprehensive study.

## 4. Patients and Methods

### 4.1. COVID-19 Patients and Samples

We performed a prospective analysis of nasopharyngeal swabs (NPS) and blood (BL) samples collected from 18 COVID-19 patients infected between December 2020 and April 2021. The patients were all recruited in the acute phase of COVID-19, during the 7-day period from the onset of the disease. Among 18 patients, 12 were hospitalized but with different disease severity, and 6 non-hospitalized individuals were tested due to mild symptoms and suspicion of COVID-19. Hospitalized patients were treated at the University Hospital Center Dr Dragisa Misovic, Belgrade, and the Serbian Institute of Occupational Health, Belgrade. Patients with mild symptoms were tested on request in the Laboratory of Molecular Microbiology, Institute for Biocides and Medical Ecology, Belgrade.

Both NPS and BL samples were collected from every participant immediately after inclusion in the study, and they were defined as “cases.” Their samples were paired during the second sampling (nasopharyngeal swab and blood) after resolving the SARS-CoV-2 infection (2–3 weeks after initial recruiting and sampling), and then they were defined as “controls”. At the moment of the first sampling, according to the set inclusion criteria, nasopharyngeal swabs all tested positive for SARS-CoV-2 RNA by Real-Time PCR. On the other hand, paired nasopharyngeal swabs taken after resolving the acute infection all tested negative for SARS-CoV-2 RNA by the same methodology.

### 4.2. RNA Extraction and Real-Time PCR

RNA isolation and SARS-CoV-2 detection was performed in the Laboratory of Molecular Microbiology, Institute for Biocides and Medical Ecology, Belgrade. RNA extraction from 200 μL of whole blood or 500 μL of viral transport medium, in which nasopharyngeal swabs were placed, was carried out using RNeasy Mini Kit (Qiagen, Kit Cat. No. 74104). Real-time PCR to confirm SARS-CoV-2 positivity was routinely performed from 5 μL of eluted RNA using GeneFinderTM COVID-19 Plus RealAmp Kit (OSANG Healthcare Co., Seongnam, Republic of Korea) following the manufacturer’s protocol. Quant StudioTM 5 Real-Time PCR Instrument (Thermo Fisher Scientific, Waltham, MA, USA) was used for the amplification and detection of viral RNA. All eluates were stored at −80 °C until shipment to the other laboratory for the NGS analysis.

### 4.3. Sequencing

Commercial sequencing was carried out at Novogene Bioinformatics Technology Co., Ltd., in Beijing, China. Messenger RNA was purified from total RNA using poly-T oligo-attached magnetic beads after rRNA removal (Illumina, Kit Cat. No. 20020597). After fragmentation, the first strand of cDNA was synthesized using random hexamer primers, followed by the second strand of cDNA synthesis (Illumina, Kit Cat. No. 20020597). The library was ready after end repair, A-tailing, adapter ligation, size selection, amplification, and purification. Paired-end sequencing of 150 bases each was performed on Illumina NovaSeq 6000. QC was done according to the sequencing provider’s specification, and 6 samples of blood with their pairs were removed from the blood batch of samples. The total number of paired samples was 18 pairs of nasopharyngeal swabs and 12 samples of blood. The average base quality Phred score was above 30 at all locations in all samples.

### 4.4. Bioinformatic Processing

Both nasopharyngeal and blood samples are quantified in an identical manner using Kallisto 0.44 [49] for pseudo-alignment to Gencode v35 transcriptome [50]. The internal probabilistic model assigns reads that do not have a transcript-level unique mapping with maximum likelihood providing abundance estimated (transcripts-per-million). Raw counts per transcript were summed at the gene level. Batch effects were explored and combated using SVA R-package [51]. The number of estimated surrogate variables by the Leek method was one, and it did not affect the results. This indicates no significant batch effects. All the samples were verified to have at least 10% expressed transcripts (non-zero values), and all the transcripts that had less than one count in more than 90% of samples rounded were removed. Differentially expressed gene (DEG) analysis was performed using EdgeR [52], and the normalization of libraries was performed using the TMM method. To test the effect of pairing, each analysis is repeated with and without the subject ID as a covariate; no additional covariates were used. The default FDR multiple testing correction level of 0.05 was used to assess statistical significance. All significant genes and their fold change were used for discovering affected gene sets and pathways. Using Panther.db with three separate GO datasets (molecular function, cellular components, biological processes). The input was the list of genes with their fold changes from EdgeR; the referent gene set was the built-in complete set of human genes. The chosen test was statistical enrichment based on Mann-Whitney U-test to utilize the numerical data available.

The significantly affected genes from DEG analysis were separated into two gene sets according to the direction of change and analyzed with the *L1000CDS2* web service to find the chemical perturbagens with the expression pattern opposite to that of our disease. The direction of change was taken into account without the numerical values for easier interpretation of the output. Genes without an HGNC name necessary for this step were manually added from the latest version of the Ensembl database. Combinations of two perturbagens were also included in the outputs. The ranking score used was the overlap ratio, and top-scoring hits were visualized with web Clustergrammer.

TPM results from Kallisto quantification were pooled and processed for the detection of alternative splicing (AS) changes using the tool Suppa2 [53]. To match the version, both isoforms and splicing events are quantified with Gencode v35 GTF using the empirical method for *p*-value calculation without using a delta PSI threshold. Due to the empirical nature of the calculation and lack of linear models, the samples were treated as unpaired (i.e., sample pairing was not accounted for).

Metagenomic analysis was performed by aligning all the samples read-wise to the joint human-bacteria-virus-SARS-CoV-2 reference using a centrifuge. The numbered of mapped reads was scaled with the total number of reads in the sample. The difference in medians between case and control samples was performed using the median test that ignores pairing. The median test was used instead of the Wilcoxon sign rank due to the immunity to a large number of ties in most samples. Multiple testing correction was performed using the Bonferroni method. All 95 columns with different assemblies of SARS-CoV-2 were summed and log-transformed, and the difference in abundances was tested using the t-test for dependent samples.

## Figures and Tables

**Table 1:** Results of paired DEG analysis in blood (BL) showing genes with FDR corrected significant *p*-value.

Gene_Name	logFC	logCPM	LR	*p*-Value	FDR	Ensembl_ID
*IFI27*	4.48	6.73	136.83	1.31 × 10^−31^	7.47 × 10^−27^	ENSG00000165949.12
*SFT2D3*	−7.48	0.76	64.85	8.07 × 10^−16^	2.30 × 10^−11^	ENSG00000173349.6
*IGLC1*	2.91	3.08	40.56	1.91 × 10^−10^	2.90 × 10^−6^	ENSG00000211675.2
*IGKV4-1*	2.14	3.49	40.43	2.04 × 10^−10^	2.90 × 10^−6^	ENSG00000211598.2
*JCHAIN*	2.42	6.87	36.21	1.77 × 10^−9^	1.64 × 10^−5^	ENSG00000132465.12
*H3C3*	1.64	2.81	36.11	1.87 × 10^−9^	1.64 × 10^−5^	ENSG00000287080.2
*AC118281.1*	−4.66	0.03	35.96	2.02 × 10^−9^	1.64 × 10^−5^	ENSG00000288380.1
*AC020656.1*	6.68	0.55	35.55	2.48 × 10^−9^	1.77 × 10^−5^	ENSG00000257764.2
*IGLV3-19*	3.92	3.72	33.73	6.33 × 10^−9^	4.01 × 10^−5^	ENSG00000211663.2
*HIST1H2BO*	1.72	2.89	33	9.24 × 10^−9^	5.26 × 10^−5^	ENSG00000274641.2
*HIST1H2BH*	1.56	3.61	31.66	1.83 × 10^−8^	9.50 × 10^−5^	ENSG00000275713.2
*IGKV1-5*	1.81	3.37	31.37	2.13 × 10^−8^	1.01 × 10^−4^	ENSG00000243466.1
*IGLC3*	2.56	5.26	30.42	3.48 × 10^−8^	1.53 × 10^−4^	ENSG00000211679.2
*HIST1H2AJ*	1.69	3.44	29.37	5.99 × 10^−8^	2.28 × 10^−4^	ENSG00000276368.2
*IGKV3-20*	1.96	3.94	29.36	6.00 × 10^−8^	2.28 × 10^−4^	ENSG00000239951.1
*AC114546.3*	−5.71	−0.81	28.95	7.44 × 10^−8^	2.62 × 10^−4^	ENSG00000279364.1
*IGHV3-30*	2.03	4.03	28.83	7.88 × 10^−8^	2.62 × 10^−4^	ENSG00000270550.1
*IGHV4-34*	2.77	3.72	28.74	8.29 × 10^−8^	2.62 × 10^−4^	ENSG00000211956.2
*IGKC*	2.06	7.21	28.02	1.20 × 10^−7^	3.60 × 10^−4^	ENSG00000211592.8
*HIST1H3F*	1.48	3.1	27.61	1.49 × 10^−7^	4.22 × 10^−4^	ENSG00000277775.2
*IGLC2*	2.56	6.3	27.52	1.56 × 10^−7^	4.22 × 10^−4^	ENSG00000211677.2
*IGKV3-15*	1.54	2.4	27.2	1.84 × 10^−7^	4.75 × 10^−4^	ENSG00000244437.1
*IGHA1*	1.77	6.28	26.75	2.32 × 10^−7^	5.74 × 10^−4^	ENSG00000211895.5
*IGLV1-40*	2.56	1.93	25.53	4.36 × 10^−7^	1.03 × 10^−3^	ENSG00000211653.2
*HIST1H2BE*	1.21	3.94	25.02	5.68 × 10^−7^	1.29 × 10^−3^	ENSG00000274290.3
*PVRIG2P*	−5.22	−1.02	24.62	7.00 × 10^−7^	1.53 × 10^−3^	ENSG00000235333.3
*IGLV3-21*	2.28	2.7	24.23	8.54 × 10^−7^	1.80 × 10^−3^	ENSG00000211662.2
*IGHV3-23*	1.53	3.26	24.03	9.48 × 10^−7^	1.93 × 10^−3^	ENSG00000211949.3
*TXNDC5*	1.7	6.72	23.76	1.09 × 10^−6^	2.14 × 10^−3^	ENSG00000239264.9
*HIST1H2BB*	1.61	1.59	23.39	1.32 × 10^−6^	2.51 × 10^−3^	ENSG00000276410.4
*IGHV3-11*	2.14	1.28	23.18	1.47 × 10^−6^	2.71 × 10^−3^	ENSG00000211941.3
*HIST1H2BM*	1.24	2.42	22.22	2.43 × 10^−6^	4.32 × 10^−3^	ENSG00000273703.2
*HIST1H2BI*	1.47	2.93	21.75	3.11 × 10^−6^	5.37 × 10^−3^	ENSG00000278588.2
*EPSTI1*	1.92	7.56	21.42	3.69 × 10^−6^	6.18 × 10^−3^	ENSG00000133106.14
*TIGD3*	−1.19	2.79	21.23	4.08 × 10^−6^	6.57 × 10^−3^	ENSG00000173825.7
*IGHG1*	2.04	5.83	21.19	4.15 × 10^−6^	6.57 × 10^−3^	ENSG00000211896.7
*IGHV3-21*	1.71	2.49	21.06	4.44 × 10^−6^	6.83 × 10^−3^	ENSG00000211947.2
*HIST1H3G*	1.3	3.63	20.9	4.84 × 10^−6^	7.25 × 10^−3^	ENSG00000273983.1
*HIST1H4L*	1.2	1.95	20.58	5.72 × 10^−6^	8.35 × 10^−3^	ENSG00000275126.2
*IGKV1D-39*	1.67	3.07	20.43	6.18 × 10^−6^	8.79 × 10^−3^	ENSG00000251546.1
*AC104837.2*	2.18	0.84	20.28	6.68 × 10^−6^	9.20 × 10^−3^	ENSG00000238015.2
*HIST1H1T*	1.54	1.27	20.25	6.78 × 10^−6^	9.20 × 10^−3^	ENSG00000187475.6
*IGKV1-12*	2.18	1.38	19.9	8.16 × 10^−6^	1.08 × 10^−2^	ENSG00000243290.3
*IGLV6-57*	2.26	1.22	19.81	8.57 × 10^−6^	1.11 × 10^−2^	ENSG00000211640.4
*IGKV3-11*	1.82	3.08	19.26	1.14 × 10^−5^	1.44 × 10^−2^	ENSG00000241351.3
*HIST1H1B*	1.42	5.57	18.96	1.33 × 10^−5^	1.65 × 10^−2^	ENSG00000184357.5
*AL031777.3*	1.1	4.35	18.9	1.38 × 10^−5^	1.67 × 10^−2^	ENSG00000282988.2
*HIST1H4D*	1.32	3.92	18.73	1.51 × 10^−5^	1.79 × 10^−2^	ENSG00000277157.2
*IGHV3-64D*	2.31	0.26	18.57	1.64 × 10^−5^	1.90 × 10^−2^	ENSG00000282639.1
*IGLV2-8*	1.96	2.03	18.53	1.67 × 10^−5^	1.90 × 10^−2^	ENSG00000278196.3
*TNFRSF17*	1.55	1.62	18.48	1.72 × 10^−5^	1.92 × 10^−2^	ENSG00000048462.11
*AC093010.3*	−3.53	1.2	18.44	1.76 × 10^−5^	1.93 × 10^−2^	ENSG00000259976.3
*IGLV3-25*	2.84	2.92	18.32	1.87 × 10^−5^	2.01 × 10^−2^	ENSG00000211659.2
*IGHV1-2*	1.7	2.01	18.23	1.96 × 10^−5^	2.06 × 10^−2^	ENSG00000211934.3
*HIST1H2AB*	1.23	2.32	18.18	2.01 × 10^−5^	2.08 × 10^−2^	ENSG00000278463.2
*PDZK1IP1*	−1.62	4.52	17.91	2.32 × 10^−5^	2.36 × 10^−2^	ENSG00000162366.8
*JUP*	1.06	3.66	17.85	2.39 × 10^−5^	2.37 × 10^−2^	ENSG00000173801.17
*IGHV5-51*	1.52	2.46	17.83	2.41 × 10^−5^	2.37 × 10^−2^	ENSG00000211966.2
*HIST1H2BD*	1.4	5.26	17.8	2.45 × 10^−5^	2.37 × 10^−2^	ENSG00000158373.8
*AC139495.1*	1.16	4.12	17.47	2.92 × 10^−5^	2.77 × 10^−2^	ENSG00000248477.6
*IGHV4-59*	1.63	3.15	17.2	3.36 × 10^−5^	3.14 × 10^−2^	ENSG00000224373.3
*IFI44L*	2.34	8.64	17.17	3.42 × 10^−5^	3.14 × 10^−2^	ENSG00000137959.16
*AC007556.1*	1.95	0.23	16.98	3.78 × 10^−5^	3.42 × 10^−2^	ENSG00000235321.1
*HIST1H4I*	1.01	3.33	16.74	4.29 × 10^−5^	3.73 × 10^−2^	ENSG00000276180.1
*HIST1H1PS1*	1.36	1.62	16.73	4.30 × 10^−5^	3.73 × 10^−2^	ENSG00000216331.2
*LY6E*	1.33	6.11	16.72	4.32 × 10^−5^	3.73 × 10^−2^	ENSG00000160932.11
*IGHV4-31*	1.86	1.5	16.51	4.84 × 10^−5^	4.11 × 10^−2^	ENSG00000231475.3
*AC016993.1*	−4.03	−1.48	16.47	4.93 × 10^−5^	4.11 × 10^−2^	ENSG00000258178.1
*HIST1H2BG*	1.23	4.05	16.42	5.07 × 10^−5^	4.11 × 10^−2^	ENSG00000273802.2
*HIST1H2AH*	1.21	3.21	16.41	5.10 × 10^−5^	4.11 × 10^−2^	ENSG00000274997.2
*IGLV2-14*	1.29	3.45	16.4	5.13 × 10^−5^	4.11 × 10^−2^	ENSG00000211666.2
*IFI44*	2.15	7.31	16.3	5.40 × 10^−5^	4.26 × 10^−2^	ENSG00000137965.11
*IGLV2-11*	1.53	1.73	16.28	5.46 × 10^−5^	4.26 × 10^−2^	ENSG00000211668.2
*RF00019*	1.38	1.59	16.23	5.61 × 10^−5^	4.30 × 10^−2^	ENSG00000207117.1
*SIGLEC1*	2.52	6.38	16.21	5.66 × 10^−5^	4.30 × 10^−2^	ENSG00000088827.12
*HIST1H3J*	1.19	2.85	16.09	6.03 × 10^−5^	4.47 × 10^−2^	ENSG00000197153.5
*IGHG2*	1.14	3.98	16.09	6.04 × 10^−5^	4.47 × 10^−2^	ENSG00000211893.4
*AC245128.3*	4.59	−1.21	15.98	6.40 × 10^−5^	4.67 × 10^−2^	ENSG00000268734.1

LogFC—log-fold-change, logCPM—log counts per million, LR—likelihood ratio.

**Table 2:** Results of paired DEG analysis in nasopharyngeal swabs (NPS) showing genes with FDR corrected significant *p*-value.

Gene Name	logFC	logCPM	LR	*p*-Value	FDR	Ensembl_ID
*ISG15*	4.76	8.21	56.21	6.51 × 10^−14^	2.26 × 10^−9^	ENSG00000187608.10
*IFITM1*	4.21	7.00	48.81	2.83 × 10^−12^	4.91 × 10^−8^	ENSG00000185885.16
*IRF7*	5.53	5.15	41.16	1.40 × 10^−10^	1.63 × 10^−6^	ENSG00000185507.21
*CXCL11*	7.02	4.02	40.48	1.99 × 10^−10^	1.73 × 10^−6^	ENSG00000169248.13
*IFIT2*	4.95	5.55	35.61	2.41 × 10^−9^	1.67 × 10^−5^	ENSG00000119922.10
*C1QB*	5.26	4.40	28.84	7.85 × 10^−8^	4.55 × 10^−4^	ENSG00000173369.17
*IFITM3*	3.07	7.10	28.01	1.21 × 10^−7^	5.99 × 10^−4^	ENSG00000142089.16
*CXCL10*	6.01	4.70	27.17	1.86 × 10^−7^	8.09 × 10^−4^	ENSG00000169245.6
*PSAP*	3.34	6.67	26.35	2.84 × 10^−7^	1.10 × 10^−^³	ENSG00000197746.14
*IFI30*	2.69	6.70	22.43	2.18 × 10^−6^	7.38 × 10^−^³	ENSG00000216490.4
*IFI35*	3.79	4.86	22.30	2.34 × 10^−6^	7.38 × 10^−^³	ENSG00000068079.8
*EPSTI1*	4.37	4.04	21.38	3.78 × 10^−6^	1.09 × 10^−2^	ENSG00000133106.14
*IL10RA*	5.83	3.15	21.11	4.33 × 10^−6^	1.16 × 10^−2^	ENSG00000110324.11
*FCGR3A*	4.93	3.61	20.73	5.28 × 10^−6^	1.31 × 10^−2^	ENSG00000203747.12
*IFIT1*	2.72	6.27	20.50	5.97 × 10^−6^	1.38 × 10^−2^	ENSG00000185745.10
*LAG3*	5.92	2.88	20.36	6.43 × 10^−6^	1.40 × 10^−2^	ENSG00000089692.9
*LAP3*	3.14	5.75	20.04	7.58 × 10^−6^	1.55 × 10^−2^	ENSG00000002549.12
*HLA-B*	2.61	7.07	19.75	8.80 × 10^−6^	1.59 × 10^−2^	ENSG00000234745.11
*IFI6*	2.37	7.08	19.67	9.20 × 10^−6^	1.59 × 10^−2^	ENSG00000126709.15
*BST2*	3.17	5.49	19.60	9.55 × 10^−6^	1.59 × 10^−2^	ENSG00000130303.13
*AC020763.4*	5.83	3.06	19.54	9.87 × 10^−6^	1.59 × 10^−2^	ENSG00000279569.1
*IFI27*	2.46	7.93	19.48	1.01 × 10^−5^	1.59 × 10^−2^	ENSG00000165949.12
*DHX58*	5.03	3.70	19.42	1.05 × 10^−5^	1.59 × 10^−2^	ENSG00000108771.13
*NAPSB*	4.43	3.74	19.30	1.12 × 10^−5^	1.62 × 10^−2^	ENSG00000131401.11
*OS9*	3.61	4.86	19.06	1.27 × 10^−5^	1.77 × 10^−2^	ENSG00000135506.16
*MNDA*	3.80	4.90	18.58	1.63 × 10^−5^	1.96 × 10^−2^	ENSG00000163563.8
*HLA-DRA*	2.84	6.65	18.55	1.65 × 10^−5^	1.96 × 10^−2^	ENSG00000204287.14
*LY6E*	2.32	7.05	18.54	1.67 × 10^−5^	1.96 × 10^−2^	ENSG00000160932.11
*GADD45B*	3.46	4.94	18.47	1.72 × 10^−5^	1.96 × 10^−2^	ENSG00000099860.9
*ISG20*	2.90	5.45	18.46	1.74 × 10^−5^	1.96 × 10^−2^	ENSG00000172183.15
*FOS*	2.91	5.60	18.45	1.75 × 10^−5^	1.96 × 10^−2^	ENSG00000170345.10
*MRPS34*	3.77	4.84	18.25	1.93 × 10^−5^	2.10 × 10^−2^	ENSG00000074071.15
*HLA-A*	2.37	6.52	18.07	2.13 × 10^−5^	2.25 × 10^−2^	ENSG00000206503.13
*MT-ND6*	2.44	11.17	17.79	2.47 × 10^−5^	2.52 × 10^−2^	ENSG00000198695.2
*ERP44*	3.52	4.57	17.55	2.79 × 10^−5^	2.77 × 10^−2^	ENSG00000023318.8
*GZMB*	5.14	3.65	17.13	3.48 × 10^−5^	3.36 × 10^−2^	ENSG00000100453.13
*HLA-DPA1*	2.93	5.63	16.68	4.41 × 10^−5^	4.15 × 10^−2^	ENSG00000231389.7
*TNFAIP6*	5.38	2.27	16.59	4.63 × 10^−5^	4.23 × 10^−2^	ENSG00000123610.5
*IL4I1*	3.84	3.94	16.26	5.52 × 10^−5^	4.92 × 10^−2^	ENSG00000104951.16
*CMPK2*	4.18	4.17	16.20	5.69 × 10^−5^	4.95 × 10^−2^	ENSG00000134326.11

LogFC—log-fold-change, logCPM—log counts per million, LR—likelihood ratio.

**Table 3:** Molecular function pathways for blood (BL) samples.

GO Molecular Function Complete	Number	Over/Under	*p*-Value	FDR
immunoglobulin receptor binding (GO:0034987)	72	+	0.00	0.00
olfactory receptor activity (GO:0004984)	369	−	0.00	0.00
antigen binding (GO:0003823)	127	+	0.00	0.00
G protein-coupled receptor activity (GO:0004930)	615	−	1.11 × 10^−16^	7.02 × 10^−14^
transmembrane signaling receptor activity (GO:0004888)	698	−	6.00 × 10^−15^	3.03 × 10^−12^
molecular transducer activity (GO:0060089)	745	−	6.46 × 10^−14^	2.33 × 10^−11^
signaling receptor activity (GO:0038023)	745	−	6.46 × 10^−14^	2.72 × 10^−11^
odorant binding (GO:0005549)	102	−	2.78 × 10^−11^	8.79 × 10^−9^
RNA binding (GO:0003723)	295	+	9.79 × 10^−10^	2.75 × 10^−7^
structural constituent of chromatin (GO:0030527)	54	+	2.92 × 10^−9^	7.39 × 10^−7^
nucleic acid binding (GO:0003676)	951	+	1.78 × 10^−7^	4.10 × 10^−5^
structural constituent of ribosome (GO:0003735)	45	+	2.27 × 10^−6^	4.79 × 10^−4^
catalytic activity. acting on a nucleic acid (GO:0140640)	91	+	3.31 × 10^−6^	6.44 × 10^−4^
heterocyclic compound binding (GO:1901363)	1311	+	5.85 × 10^−6^	1.06 × 10^−^³
organic cyclic compound binding (GO:0097159)	1332	+	9.36 × 10^−6^	1.58 × 10^−^³
oxygen binding (GO:0019825)	13	−	4.05 × 10^−5^	6.40 × 10^−^³
heme binding (GO:0020037)	36	−	1.17 × 10^−4^	1.75 × 10^−2^
catalytic activity. acting on RNA (GO:0140098)	67	+	1.34 × 10^−4^	1.89 × 10^−2^
protein binding (GO:0005515)	3412	+	1.84 × 10^−4^	2.45 × 10^−2^
oxygen carrier activity (GO:0005344)	8	−	2.41 × 10^−4^	3.05 × 10^−2^

**Table 4:** Predicted overlap of L1000 genes with individual perturbagen signatures of the opposite direction. All the differentially expressed genes are included in the analysis. Full table with links is given in Supplementary Table S10.

Rank	Score	Perturbation	Cell-Line	Dose	Time
1	0.4706	BML-259	DV90	80.0 um	6.0 h
2	0.4118	BRD-K12184916	SNGM	0.63 um	6.0 h
3	0.3529	Dilazep dihydrochloride	A549	10.0 um	6.0 h
4	0.3529	16-HYDROXYTRIPTOLIDE	AGS	0.08 um	6.0 h
5	0.3529	TG101348	DV90	11.1 um	6.0 h
6	0.3529	HLI 373	SNGM	10.0 um	6.0 h
7	0.3529	528116.cdx	SNGM	0.09 um	6.0 h
8	0.3529	BRD-K68548958	SNGM	20.0 um	6.0 h
9	0.3529	N9-isoproplyolomoucine	TYKNU	122.55 um	6.0 h
10	0.3529	BRD-K68756823	VCAP	10.0 um	24.0 h
11	0.3529	vorinostat	HCC515	3.33 um	24 h
12	0.2941	TG101348	NCIH2073	11.1 um	6.0 h
13	0.2941	528116.cdx	NCIH2073	0.09 um	6.0 h
14	0.2941	curcumin	SNGM	48.0 um	6.0 h
15	0.2941	MK-0591	U937	80.0 um	6.0 h
16	0.2941	chaetocin	VCAP	0.08 um	6.0 h
17	0.2941	NCGC00185090-03	HEPG2	10.0 um	6.0 h
18	0.2941	BRD-K23657553	SKB	10.0 um	24.0 h
19	0.2941	BRD-K63945320	ASC	10.0 um	24.0 h
20	0.2941	BRD-K11927976	HEPG2	10.0 um	6.0 h
21	0.2941	BRD-K48692744	A549	10.0 um	24.0 h
22	0.2941	BRD-K63606607	MCF7	10.0 um	6.0 h
23	0.2941	ZM-447439	MCF7	3.33 um	24 h
24	0.2941	PD-0325901	HEPG2	10 um	24 h
25	0.2941	GSK-461364	HEPG2	0.12 um	24 h
26	0.2941	KU-60019	HEPG2	10 um	24 h
27	0.2941	torin-2	SKBR3	10 um	24 h
28	0.2941	torin-2	SKBR3	3.33 um	24 h
29	0.2941	NU-7441	SKBR3	10 um	3 h
30	0.2941	torin-2	SKBR3	10 um	3 h
31	0.2941	GSK-2126458	MCF7	3.33 um	3 h
32	0.2941	QL-XII-47	SKBR3	10 um	24 h
33	0.2941	pazopanib	HCC515	3.33 um	24 h
34	0.2353	NOCODAZOLE	HA1E	10.0 um	24.0 h
35	0.2353	CYCLOHEXIMIDE	VCAP	10.0 um	6.0 h
36	0.2353	CARBAMAZEPINE	VCAP	10.0 um	6.0 h
37	0.2353	CGS 15943	MCF7	10.0 um	6.0 h
38	0.2353	N9-isoproplyolomoucine	A549	122.55 um	6.0 h
39	0.2353	PROSTAGLANDIN A1	A673	1.0 um	6.0 h
40	0.2353	PI 103 hydrochloride	A673	11.1 um	6.0 h
41	0.2353	AG14361	AGS	25.0 um	6.0 h
42	0.2353	COT-10b	AGS	44.4 um	6.0 h
43	0.2353	AG14361	CORL23	25.0 um	6.0 h
44	0.2353	Narciclasine	CORL23	10.0 um	6.0 h
45	0.2353	16-HYDROXYTRIPTOLIDE	DV90	0.08 um	6.0 h
46	0.2353	QUINACRINE HYDROCHLORIDE	DV90	10.0 um	6.0 h
47	0.2353	KU 0.060648 trihydrochloride	DV90	10.0 um	6.0 h
48	0.2353	COT-10b	DV90	44.4 um	6.0 h
49	0.2353	PNU 74654	DV90	80.0 um	6.0 h
50	0.2353	BRD-K68548958	H1299	20.0 um	6.0 h

**Table 5:** Predicted overlap of L1000 genes with signatures of gene pairs of the opposite direction. As opposed to the previous table, the histone genes are excluded from the comparison. The highest overlap, as represented by the score, is 64.71%.

Rank	Score	Combination	
1	0.6471	2. BRD-K12184916	34. NOCODAZOLE
2	0.5882	1. BML-259	2. BRD-K12184916
3	0.5882	1. BML-259	3. Dilazep dihydrochloride
4	0.5882	1. BML-259	9. N9-isoproplyolomoucine
5	0.5882	1. BML-259	19. BRD-K63945320
6	0.5882	3. Dilazep dihydrochloride	34. NOCODAZOLE
7	0.5882	4. 16-HYDROXYTRIPTOLIDE	34. NOCODAZOLE
8	0.5882	5. TG101348	34. NOCODAZOLE
9	0.5882	6. HLI 373	34. NOCODAZOLE
10	0.5882	7. 528116.cdx	34. NOCODAZOLE
11	0.5882	10. BRD-K68756823	34. NOCODAZOLE
12	0.5882	11. vorinostat	34. NOCODAZOLE
13	0.5294	1. BML-259	4. 16-HYDROXYTRIPTOLIDE
14	0.5294	1. BML-259	5. TG101348
15	0.5294	1. BML-259	6. HLI 373
16	0.5294	1. BML-259	7. 528116.cdx
17	0.5294	1. BML-259	8. BRD-K68548958
18	0.5294	1. BML-259	10. BRD-K68756823
19	0.5294	1. BML-259	11. vorinostat
20	0.5294	1. BML-259	12. TG101348
21	0.5294	1. BML-259	16. chaetocin
22	0.5294	2. BRD-K12184916	17. NCGC00185090-03
23	0.5294	1. BML-259	18. BRD-K23657553
24	0.5294	2. BRD-K12184916	19. BRD-K63945320
25	0.5294	1. BML-259	20. BRD-K11927976
26	0.5294	2. BRD-K12184916	21. BRD-K48692744
27	0.5294	1. BML-259	23. ZM-447439
28	0.5294	2. BRD-K12184916	23. ZM-447439
29	0.5294	1. BML-259	24. PD-0325901
30	0.5294	2. BRD-K12184916	25. GSK-461364
31	0.5294	1. BML-259	26. KU-60019
32	0.5294	1. BML-259	27. torin-2
33	0.5294	1. BML-259	28. torin-2
34	0.5294	1. BML-259	30. torin-2
35	0.5294	1. BML-259	31. GSK-2126458
36	0.5294	1. BML-259	32. QL-XII-47
37	0.5294	1. BML-259	34. NOCODAZOLE
38	0.5294	8. BRD-K68548958	34. NOCODAZOLE
39	0.5294	9. N9-isoproplyolomoucine	34. NOCODAZOLE
40	0.5294	12. TG101348	34. NOCODAZOLE
41	0.5294	13. 528116.cdx	34. NOCODAZOLE
42	0.5294	14. curcumin	34. NOCODAZOLE
43	0.5294	16. chaetocin	34. NOCODAZOLE
44	0.5294	18. BRD-K23657553	34. NOCODAZOLE
45	0.5294	20. BRD-K11927976	34. NOCODAZOLE
46	0.5294	22. BRD-K63606607	34. NOCODAZOLE
47	0.5294	24. PD-0325901	34. NOCODAZOLE
48	0.5294	26. KU-60019	34. NOCODAZOLE
49	0.5294	27. torin-2	34. NOCODAZOLE
50	0.5294	28. torin-2	34. NOCODAZOLE

**Table 6:** Overview of alternatively spliced events and isoform across nasopharyngeal swabs (NPS) and blood (BL) samples.

Tissue	Type	ID	dPSI	*p*-Value	Gene
Nasal	Event	ENSG00000137502.10;SE:chr11:82987770-82994039:82994122-82997224:−	0.2929	0.0475	*RAB30*
		ENSG00000188215.10;AF:chr16:20862643-20868895:20868980:20862643-20900204:20900358:−	−0.0801	0.0490	*DCUN1D3*
		ENSG00000198089.16;AF:chr22:31604314:31604404-31604869:31604569:31604671-31604869:+	−0.1146	0.0485	*SFI1*
		ENSG00000271503.6;SE:chr17:35872464-35875561:35875642-35878528:−	0.2111	0.0470	*CCL5*
	Isoform	ENST00000599180.2	0.0977	0.0345	*FFAR2*
		ENST00000601590.1	−0.1032	0.0345	*FFAR2*
		ENST00000324873.8	−0.4075	0.0485	*NUPR1*
		ENST00000395641.2	0.2033	0.0485	*NUPR1*
		ENST00000567646.1	0.2043	0.0485	*NUPR1*
		ENST00000324344.9	0.0801	0.0255	*DCUN1D3*
		ENST00000563934.1	−0.0801	0.0255	*DCUN1D3*
Blood	Event	ENSG00000115993.13;AF:chr2:201420706-201433464:201433554:201420706-201451350:201451458:−	0.2195	0.0470	*TRAK2*
		ENSG00000115993.13;AF:chr2:201420706-201433464:201433554:201420706-201451350:201451500:−	0.2052	0.0470	*TRAK2*
		ENSG00000205542.11;RI:chrX:12975110:12975168-12976246:12976361:+	−0.0441	0.0435	*TMSB4X*
		ENSG00000251562.8;AF:chr11:65504519:65505019-65506386:65505207:65505662-65506386:+	0.1279	0.0130	*MALAT1*
	Isoform	ENST00000379359.4	0.1200	0.0450	*RGCC*
		ENST00000487837.1	−0.1200	0.0450	*RGCC*
		ENST00000393085.4	0.1020	0.0470	*MTPN*
		ENST00000435723.1	−0.1020	0.0470	*MTPN*
		ENST00000489359.1	0.2418	0.0400	*LIPA*
		ENST00000229595.6	0.1735	0.0435	*ASF1A*
		ENST00000511766.2	−0.1735	0.0435	*ASF1A*
		ENST00000252818.5	−0.0756	0.0490	*JUND*
		ENST00000600972.1	0.0756	0.0490	*JUND*
		ENST00000259456.7	0.2388	0.0115	*HEMGN*
		ENST00000616898.2	−0.2388	0.0115	*HEMGN*
		ENST00000373474.9	0.1640	0.0405	*LMX1B*
		ENST00000526117.6	−0.1889	0.0405	*LMX1B*
		ENST00000390539.2	0.3873	0.0325	*IGHA2*
		ENST00000497872.4	−0.3873	0.0325	*IGHA2*
		ENST00000390559.6	0.1967	0.0070	*IGHM*
		ENST00000637539.2	−0.1967	0.0070	*IGHM*
		ENST00000414005.1	−0.1595	0.0380	*Lnc-TMEM121-3*
		ENST00000418566.1	0.1595	0.0380	*Lnc-TMEM121-3*
		ENST00000560255.2	0.1866	0.0445	*ST20-AS1*
		ENST00000618735.1	−0.1866	0.0445	*ST20-AS1*
		ENST00000601040.1	0.1474	0.0410	*Lnc-TMEM38A-2*
		ENST00000601687.1	−0.1474	0.0410	*Lnc-TMEM38A-2*

## Data Availability

The datasets used and/or analyzed during the current study are available from the corresponding author upon reasonable request.

## Supplementary Materials

The following supporting information can be downloaded at: https://www.mdpi.com/article/10.3390/ijms241713125/s1.
